# Reliable Prediction Models Based on Enriched Data for Identifying the Mode of Childbirth by Using Machine Learning Methods: Development Study

**DOI:** 10.2196/28856

**Published:** 2021-06-04

**Authors:** Zahid Ullah, Farrukh Saleem, Mona Jamjoom, Bahjat Fakieh

**Affiliations:** 1 Department of Information Systems, Faculty of Computing and Information Technology King Abdulaziz University Jeddah Saudi Arabia; 2 Department of Computer Sciences, College of Computer and Information Sciences Princess Nourah Bint Abdulrahman University Riyadh Saudi Arabia

**Keywords:** machine learning, prediction model, health care, cesarean, delivery, decision making

## Abstract

**Background:**

The use of artificial intelligence has revolutionized every area of life such as business and trade, social and electronic media, education and learning, manufacturing industries, medicine and sciences, and every other sector. The new reforms and advanced technologies of artificial intelligence have enabled data analysts to transmute raw data generated by these sectors into meaningful insights for an effective decision-making process. Health care is one of the integral sectors where a large amount of data is generated daily, and making effective decisions based on these data is therefore a challenge. In this study, cases related to childbirth either by the traditional method of vaginal delivery or cesarean delivery were investigated. Cesarean delivery is performed to save both the mother and the fetus when complications related to vaginal birth arise.

**Objective:**

The aim of this study was to develop reliable prediction models for a maternity care decision support system to predict the mode of delivery before childbirth.

**Methods:**

This study was conducted in 2 parts for identifying the mode of childbirth: first, the existing data set was enriched and second, previous medical records about the mode of delivery were investigated using machine learning algorithms and by extracting meaningful insights from unseen cases. Several prediction models were trained to achieve this objective, such as decision tree, random forest, AdaBoostM1, bagging, and k-nearest neighbor, based on original and enriched data sets.

**Results:**

The prediction models based on enriched data performed well in terms of accuracy, sensitivity, specificity, F-measure, and receiver operating characteristic curves in the outcomes. Specifically, the accuracy of k-nearest neighbor was 84.38%, that of bagging was 83.75%, that of random forest was 83.13%, that of decision tree was 81.25%, and that of AdaBoostM1 was 80.63%. Enrichment of the data set had a good impact on improving the accuracy of the prediction process, which supports maternity care practitioners in making decisions in critical cases.

**Conclusions:**

Our study shows that enriching the data set improves the accuracy of the prediction process, thereby supporting maternity care practitioners in making informed decisions in critical cases. The enriched data set used in this study yields good results, but this data set can become even better if the records are increased with real clinical data.

## Introduction

### Background

Machine learning is increasingly prevalent in and vital to health care industries in terms of predicting and identifying quality treatments for patients and enhancing other health care services. Therefore, machine learning techniques are used for extracting knowledge from huge and complex data sets in an organized form so that it can be used for making effective decisions. According to Sana et al [1], machine learning techniques provide diagnosis and analytical amenities in several medical fields and their applications in clinical factors and analytics such as disease prediction, decision making based on extracted medical knowledge, and serving in patient management. Moreover, with the increasing amount of available data, machine learning techniques have significant benefits as prediction tools in health care [2] that sometimes provide surprising prediction models that help in clinical counseling [3]. These tools are fundamental to biomedical research and are utilized as an integral part of the clinical decision-making process [4].

Child delivery can be performed through several methods in hospitals, but the most common methods are either traditional vaginal birth or cesarean (c-section), while vacuum extractions and obstetric pincers can be used during complications in vaginal deliveries [5]. There are several assumptions pertaining to the mode of delivery, but it is still challenging to predict the type of childbirth accurately [6,7]. C-section is a technique used in maternity care for delivering children by performing a surgical incision to the woman’s abdomen and uterus [8], which normally takes place when complications arise related to the mother or a child in a normal delivery [9]. The possible complications of c-section for mothers are infections, excessive bleeding that could cause anemia, and reaction to anesthesia; therefore, maternal death rates with c-sections are higher than that for vaginal deliveries [8]. However, a c-section could be necessary to save the lives of both the mother and the child if the baby is located in a wrong position in the womb, the head of the baby is larger than the birth canal, the direction of the baby is reversed, or the mother has a c-section history or even heart-related diseases [10]. Molina et al [11] further explained that c-sections are lifesaving for obstructed labor and any other obstruction in the delivery process for decreasing baby and mother mortality, but the risk of complications and overuse can harm both mothers and babies. Every mode of delivery has its pros and cons, but selecting the wrong type may lead to a variety of risks such as baby cessation, excessive bleeding, baby breathing problems, and other similar issues [7].

The rate of c-sections is higher than the rate of normal deliveries, especially in high-income countries, where in 2012, around 23 million deliveries were conducted by c-section worldwide [11]. Prema and Pushpalatha [8] indicated that the highest rate of c-section was 29.1% in November 2005, while nearly one-third of the deliveries were conducted using c-section in 2015 [12] as reported by the Centers for Disease Control and Prevention [13]. In the United States, the c-section rate significantly increased to 60% from 1996 to 2009, and the c-section rate was 32% of all deliveries in 2007 [12]. Li et al [14] reported that in China, 46.2% of the 14,541 deliveries across 3 provinces in different hospitals were conducted by c-section in the years 2007 and 2008. Similarly, in Pakistan, around half of the total deliveries are conducted at home, but a high number of c-sections are conducted at hospitals [9]. Fergus et al [4] argued that overinterpretations increase the numbers of c-section, even if there are no specific risks involved in the normal deliveries. It is difficult to know the optimal level of the c-section rate because although the World Health Organization advocates that national rates do not exceed by 10 to 15 c-sections per 100 births, the rates of c-sections are noticeably higher [11].

### Related Work

Studies related to identifying the mode of childbirth were found in different databases such as Google Scholar, Science Direct, IEEE explorer, Wiley, ResearchGate, and other data sources. The major keywords used in the browsers were phrases such as cesarean sections using machine learning, c-sections using machine learning, machine learning in maternity care, AI in maternity care, etc. C-section is the most commonly increasing mode of delivery worldwide, and areas of concerns such as ideal c-section rate, safety, and cost are still under debate [15]. Moreover, many researchers have investigated different features to determine the main causes for cesarean delivery and have built a prediction model based on these features. Some main causes are related to the medical and obstetric history of the mother [5,6,8,15-17]. The study of Lee and Gay [18] found that sleep disturbance and fatigue in late pregnancy lead to greater chances of delivery by c-section. Others analyzed the socioeconomic or sociodemographic features [1,19] and some have determined the main causes to be the region and level of medical services afforded [9].

Wollmann et al [3] attempted to predict the chances of normal births after a c-section. In this regard, they collected data of women with one previous birth in Sweden during 2008-2014 and built 3 machine learning models and 1 regression model. They concluded that the majority of the women with a history of c-sections could still successfully deliver a baby in the normal way. Similarly, Prema and Pushpalatha [8] investigated the main causes of cesarean delivery based on the extracted features. Several machine learning models were trained on a data set collected from a pregnancy risk assessment survey. Their models have predicted c-sections with 96% accuracy for women who had a history of c-sections compared to 89% accuracy for women who had no previous c-section. Khan et al [10] presented a study to predict whether c-section is compulsory along with increased safety for both mother and child during and after delivery. They trained 3 ensemble models and found the highest accuracy model of 87.66%. They also found that for predicting the target mode of delivery, several features such as previous c-sections, amniotic fluid, fetal intrapartum pH, and preinduction should be considered. Sana et al [1] figured out the socioeconomic features that cause cesarean delivery. They trained decision tree (DT) and artificial neural network models to predict the mode of delivery in which artificial neural networks showed a high accuracy of 82%. Abbas et al [9] believed that c-section causes can be influenced by regions and therefore, they selected a region with a limited health care infrastructure. They trained several models based on 23 features in order to predict the mode of delivery, and the highest accuracy model was 91.8%. They also concluded that the maternal age and the previous mode of delivery considerably influenced the mode of the next delivery.

Ricciardi et al [20] adopted classification methods DT, random forest (RF), AdaBoostM1, gradient boosting, and DECORATE (Diverse Ensemble Creation by Oppositional Relabeling of Artificial Training Examples) for predicting patients’ mode of delivery. They applied these methods to a data set of 370 records collected from public and private hospitals from the years 2000 to 2009. RF outperformed with 91.1% accuracy, 90% sensitivity, and >96% ROC. In the study of Improta et al [2], the 4 classification methods, namely, DT, RF, AdaBoost, and gradient boosting were trained on a cardiotocographic data set for identifying the mode of delivery, in which RF showed the highest performance with 87.6% accuracy, 87.9% precision, and 93% ROC. In a study conducted by Saleem et al [21] for classifying the mode of delivery using 4 machine learning methods, the AdaBoost model showed the highest accuracy of 91.8%, sensitivity of 95.5%, and specificity of 98%. Of the 4 classification algorithms used by Pereira et al [5] to predict the mode of delivery, DT outperformed with accuracy of 84%, sensitivity of 88%, and specificity of 80%. A DT method was adopted by Soleimanian et al [15] to investigate the mode of delivery in a data set of 80 patients and they found an accuracy of 86.25%. In the study of Fergus et al [4], ensemble methods were used for classifying the mode of delivery by using a cardiotocographic tracer in which all 3 methods showed promising results of 87%, 90%, and 96% for sensitivity, specificity, and ROC, respectively. Moreover, Fergus et al [22] established that machine learning with fetal heart rate signals significantly improved the efficacy of detecting the mode of delivery compared to obstetrician and midwife predictions and other systems. Their results showed 94%, 91%, and 99% sensitivity, specificity, and ROC, respectively.

### Objective of This Study

This study aims to provide prediction models for identifying the mode of childbirth based on antenatal signs and symptoms by using machine learning techniques. To achieve the objectives of this study, the data set was first enriched with additional cases using the Synthetic Minority Oversampling Technique (SMOTE) [23]. Second, several prediction models were trained and tested on original and enriched data sets. A cross-validation of 10 folds was used for evaluating the performance of the models. In the outcomes, the enriched data set showed better performance in terms of accuracy, sensitivity, specificity, F-measure, and receiver operating characteristic (ROC) compared to the original data set. These findings encourage the applications of these models for maternity care decision support systems to predict the mode of delivery before birth.

## Methods

### Software Used

The data synthesis and analysis in terms of classifications and predicting the mode of delivery were performed using Weka software (University of Waikato, New Zealand) [24]. Weka has many machine learning algorithms that are useful for training data sets and then testing them on unseen cases to predict target values [25,26].

### Data Collection

The data set used in this study was harvested from the study of Soleimanian et al [15] and is publicly available in the University of California, Irvine machine learning repository [12,27]. The data set contains 5 features, namely, age, delivery_number, delivery_time, blood_of_pressure, and heart_problem, while cesarean is a class attribute to label whether the delivery was performed by c-section. In the data set, each attribute shows different values, such as age ranges from 22 years to 38 years, delivery_number shows the number of births from 1 to 4, delivery_time shows 3 different statuses that are premature, timely, or latecomer, blood_of_pressure also shows 3 different statuses that are low, normal, and high, the heart_problem is categorized as either yes or no, and the last attribute (cesarean) is categorized as to whether the birth was by c-section or not. This data set contains 80 records of pregnant women and information about whether delivery was conducted by c-section or normal birth.

### Data Enrichment

The data set used in this study originally contained 80 records, of which 46 records were normal vaginal deliveries while the remaining 34 were c-sections. According to the criteria of Vapnik [28], the total number of records was insufficient for predictive purposes [25]. Therefore, the data set needed more records to ensure that the prediction models are reliable and trustworthy. For this reason, the existing data set was enriched with more records using the standard method of SMOTE [23]. SMOTE is a popular method of machine learning used for oversampling [29] in which the minority class in a data set is generated by a synthetic example in the feature area based on the selected k-nearest neighbor (k-NN) from the minority class [21]. This practice has been adopted in several biomedical studies [4,30-36]. Mohammed et al [34] used the SMOTE method for enriching the minority class and concluded that oversampling has a positive impact on the prediction models. Similarly, Ramezankhani et al [32] adopted the SMOTE method for increasing the samples in the minority class in the original data set with various percentages (ie, 100%, 200%....,700%), which resulted in increased sensitivity of the different classifiers used. Another study of Hussain et al [37] used the SMOTE method and compared the results with the original data, concluding that the prediction models’ performance after oversampling was enhanced compared to the original data. According to Ebenuwa [38] and Frank [39], SMOTE can be used for increasing the size of a data set. This study used the SMOTE method for enriching the samples in both classes with 100%; therefore, the total number of records after oversampling increased to 160 while the ratio between the 2 classes remained the same as in the original data set. At the current stage, the enriched data set was sufficient for reliable prediction.

### Prediction Models

The ability to gain meaningful insight from the available unstructured and unorganized data and to utilize it as an integral part of a business decision support system is an art. There are several technologies available that work in this domain for structuring and organizing the historical data for predicting new patterns for the unseen scenarios, including machine learning. Similarly, these techniques are widely used in the health care industry, where prediction models have evolved with clinical practice in every medical field. In the literature, several studies have attempted to classify the types of childbirth from different perspectives by using machine learning models. The most widely used classifiers for predictions are DT, RF, AdaBoost, support vector machine, k-NN, Naïve Bayes, and several other techniques. This study has utilized 5 machine learning classifiers for developing prediction models that can help health care practitioners in deciding the favorable mode of delivery, primarily based on the mother’s history and condition. A brief discussion of these classifiers is presented below.

#### DT

DT is a nonparametric supervised learning technique used for both classification and regression and it uses large and complicated data sets to explore features and mined patterns that are vital to discrimination and predictive modeling. In this technique, the large data sample is divided into training and testing data sets, and based on the training data set, building a DT model and a testing data set in order to decide on the suitable tree size required to attain an optimal final model is performed [40].

#### RF

RF is an ensemble technique used for classification or regression that utilizes ﻿the input data and constructs multitude of DTs at the training time and outputs the class (classification) or the prediction mean (regression) of an individual tree [41]. In this technique, each DT is randomized using a bootstrap resampling method with random feature selection, and the classification is performed based on the voting of various randomized DTs on the final outcome [4]. Furthermore, the optimum split is computed using various feature sets and lingers until the tree is completely grown without pruning. This process is iterated for all trees in the forest by using different bootstraps of data, and the classifications of new samples are therefore based on the majority of votes cast [4].

#### AdaBoost

AdaBoost is an ensemble technique of linear member classifiers that is constructed to enhance the efficiency of the binary classifiers. In this technique, the weak learning models with better accuracy can be boosted to develop a strong prediction model. AdaBoost is an iterative-based technique where each iteration detects the misclassified data points and increases the weights of the correct points to increase the chance of the next classifiers getting them right. Moreover, in this method, the instances are moved from the iterative samples of the training data to the subsequent data set, and the classifiers are combined based on the weighted majority of votes [10,42].

#### Bagging

Bootstrap aggregation (or bagging) is an ensemble technique used for classification or regression. In bagging, a repeated sample is made from a training set by using simple random sampling with replacement, and for each bootstrap sample, a weak classifier is trained. These classifiers are then utilized for predicting class labels on testing data, and the class that obtains the majority of the votes wins [43].

#### k-NN

k-NN is a supervised learning technique that takes a data set in which the data points are labeled with different classes and uses them for learning to label the new points. The labeling of new points is based on the closest of its neighbors’ labels and the majority of votes cast; therefore, the labels of the nearest neighbors are the labels of the new points. In k-NN, k is the criteria number of checking the nearest neighbors [12,44].

### Performance Evaluation Method

There are several methods for evaluating the performance of prediction models such as using the whole data set as a training set, providing a separate test set, cross-validation, and percentage split, of which cross-validation is regarded as the most reliable method [45]. In this study, each prediction model built was evaluated using cross-validations of 10 folds [46]. In 10-fold cross-validation, the training set is divided into 10 subsets, and each subset is used once in the testing phase [47]. Amin and Ali [12] and Soleimanian et al [15] trained their models by using the whole data set as training data, but this method was not recommended in several other studies such as those of Mitchell [48], Smith and Frank [45], and Brownlee [49] because machine learning methods learn the training data and can predict them easily. As explained by Mitchell [48], utilizing the entire data set for training and testing purposes at the same time may produce unrealistic outcomes that are extremely positive and prone to overfitting. As further explained by Smith and Frank [45], the results achieved using training data as test data give rise to resubstituting errors, which are typically unjustifiably optimistic for predicting the performance of a model with future unseen data. Moreover, a training set for a model evaluation can be useful if one is more interested in a descriptive rather than a predictive model [49]. This is usually the challenge of machine learning: to predict unseen cases that have not been trained. To the contrary, cross-validation is regarded as the most profound and reliable method for model evaluation in machine learning when all data exist in 1 set [45]. Furthermore, in cross-validation, the test set contains unseen cases that are unknown to the model during the training phase, which can help reliable assessment of a classifier’s performance [50] because cross-validation helps render generalization errors and variance [51]. As further explained by Schaffer [52] cross-validation can be used to choose a classifier in case of lack of pertinent domain-specific knowledge. In short, cross-validation provides practical estimation because a model is predicting actual results that may have been unknown to the model in the training process.

## Results

This study has applied the selected prediction models to both the original data set (80 cases) and the enriched data set (160 cases). The performance of each model was evaluated using cross-validations of 10 folds [46]. In 10-fold cross-validation, the training set is divided into 10 subsets, and each subset is used once in the training phase [47]. In the implementation phase, when the required parameters are set for testing the model, a confusion matrix is calculated for each classifier run. Specifically, the confusion matrix provides 4 important values that are computed based on the correctly and incorrectly classified instances of a data set. These values are commonly known as true positive, true negative, false positive, and false negative. This matrix is the basis for calculating important measures such as model performance, model accuracy, sensitivity, specificity, and F-measure. All these measures are calculated using different equations. For example, the accuracy of a model is calculated using the following equation:


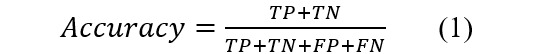


where FP=false positive, FN=false negative, TP=true positive, and TN=true negative.

**Figure 1:**
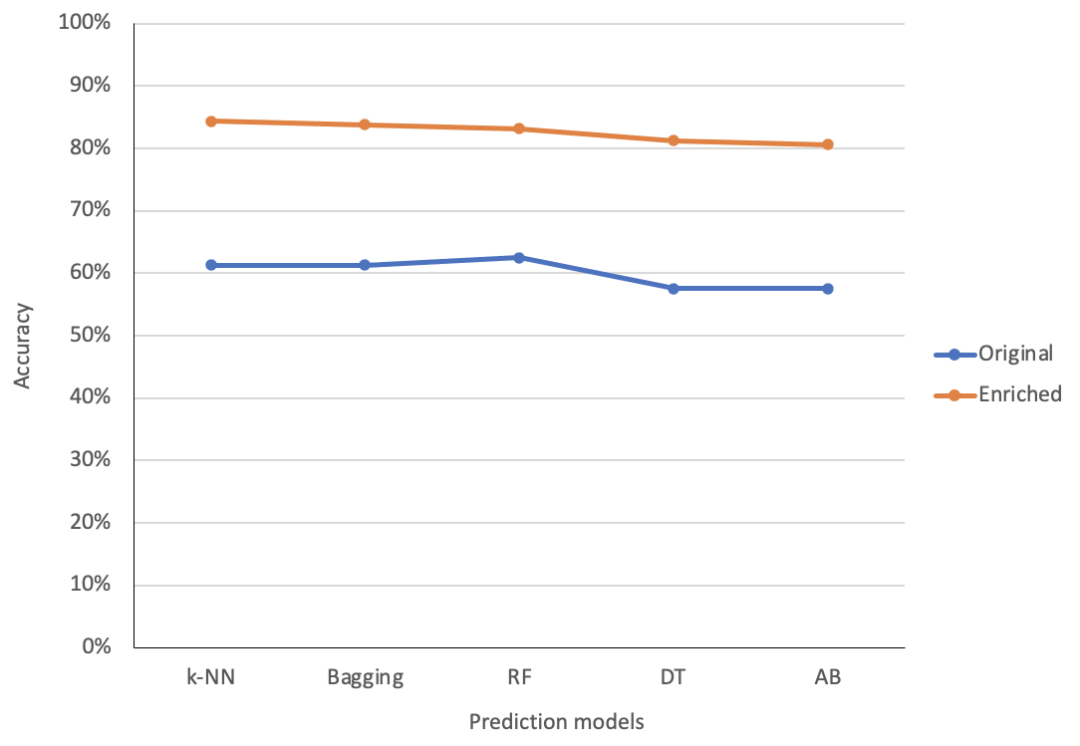
Comparison of the accuracy between models before and after data enrichment. AB: AdaBoost; DT: decision tree; k-NN: k-nearest neighbor; RF: random forest.

The accuracies of the different models before and after enriching the data set for identifying the mode of delivery were evaluated using equation 1, and the outcomes are depicted in Table 1 and Figure 1.

**Table 1:** Accuracies and kappa values of models before and after data enrichment.

Method	Original data set			Enriched data set	
	Accuracy (%)	Kappa value	Accuracy (%)		Kappa value
k-Nearest neighbor	61.25	0.228	84.38		0.685
Bagging	61.25	0.192	83.75		0.664
Random forest	62.50	0.215	83.13		0.654
Decision tree	57.50	0.181	81.25		0.612
AdaBoost	57.50	0.124	80.63		0.603

According to Table 1 and Figure 1, the performances of all the models in terms of accuracy were very low when they were trained with the original data set; however, accuracy was tremendously improved when the models were trained with the updated enriched data set, whose improvement reached approximately 20%-23%. In the original data set, RF showed the highest accuracy of 62.50%, which was far lower in performance than the lower model trained with the enriched data. Moreover, for the models trained with the enriched data set, k-NN showed the highest accuracy of 84.38%, while bagging, RF, DT, and AdaBoost showed accuracies of 83.75%, 83.13%, 81.25%, and 80.63%, respectively.

**Figure 2:**
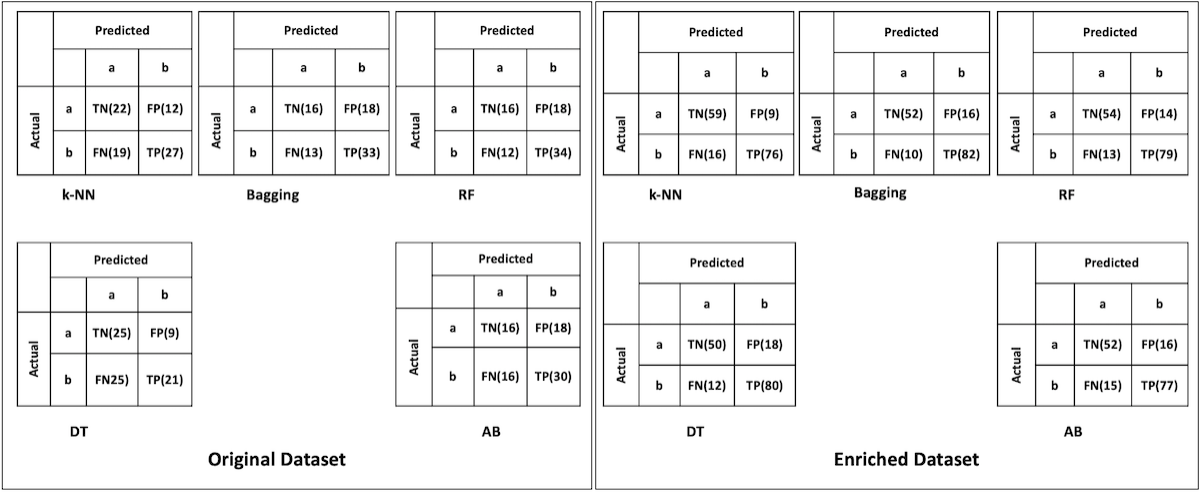
Confusion matrix of applied models before and after data enrichment. AB: AdaBoost; DT: decision tree; FN: false negative; FP: false positive; k-NN: k-nearest neighbor; RF: random forest; TN: true negative; TP: true positive.

Kappa values or kappa statistics [53] is a measure that compares the observed accuracy with the expected accuracy (random chance) and is the appropriate method when 2 or more independent classifiers are analyzing the same case [9]. There are different thresholds ranges for the kappa values [54]; however, in machine learning, when investigating an unseen scenario, a kappa value higher than 0.40 might be considered exceptional [55]. According to Table 1, the kappa values of k-NN, bagging, RF, DT, and AdaBoost in the original data set are lower than the threshold, but in the enriched data set are 0.685, 0.664, 0.654, 0.612, and 0.603, respectively, surpassing the threshold value. Moreover, Figure 2 shows the confusion matrix of the models used in this study, where “a” represents 0 class while “b” represents 1 class in the data set. Furthermore, the accuracies of the models for identifying the mode of delivery were also measured using recall, precision, and F-measure. These are the important measures computed based on the values of the confusion matrix. Recall, which is also referred to as sensitivity, is the proportion of the real positive values that are correctly classified as positive, while precision, which is referred to as predictive positive value or confidence [56] or specificity [57], is the proportion of the predicted positive values that are correctly real positives [56]. Similarly, F-measure [58] is the hormonic mean of precision and recall [59]. Table 2 and Table 3 exhibit the values of recall, precision, and F-measures for all models trained before and after data enrichment, respectively.

**Table 2:** Precision, recall, and F-measure of the models trained with original data.

Method	Cesarean section				Normal delivery		
	Precision	Recall	F-measure	Precision		Recall	F-measure
k-Nearest neighbor	0.692	0.587	0.635	0.537		0.647	0.587
Bagging	0.647	0.717	0.680	0.552		0.471	0.508
Random forest	0.654	0.739	0.694	0.571		0.471	0.516
Decision tree	0.700	0.457	0.553	0.500		0.735	0.595
AdaBoost	0.625	0.652	0.638	0.500		0.471	0.485

**Table 3:** Precision, recall, and F-measure of the models trained with enriched data.

Method	Cesarean section				Normal delivery		
	Precision	Recall	F-measure	Precision		Recall	F-measure
k-Nearest neighbor	0.894	0.826	0.859	0.787		0.868	0.825
Bagging	0.837	0.891	0.863	0.839		0.765	0.800
Random forest	0.849	0.859	0.854	0.806		0.794	0.800
Decision tree	0.816	0.870	0.842	0.806		0.735	0.769
AdaBoost	0.828	0.837	0.832	0.776		0.765	0.770

**Figure 3:**
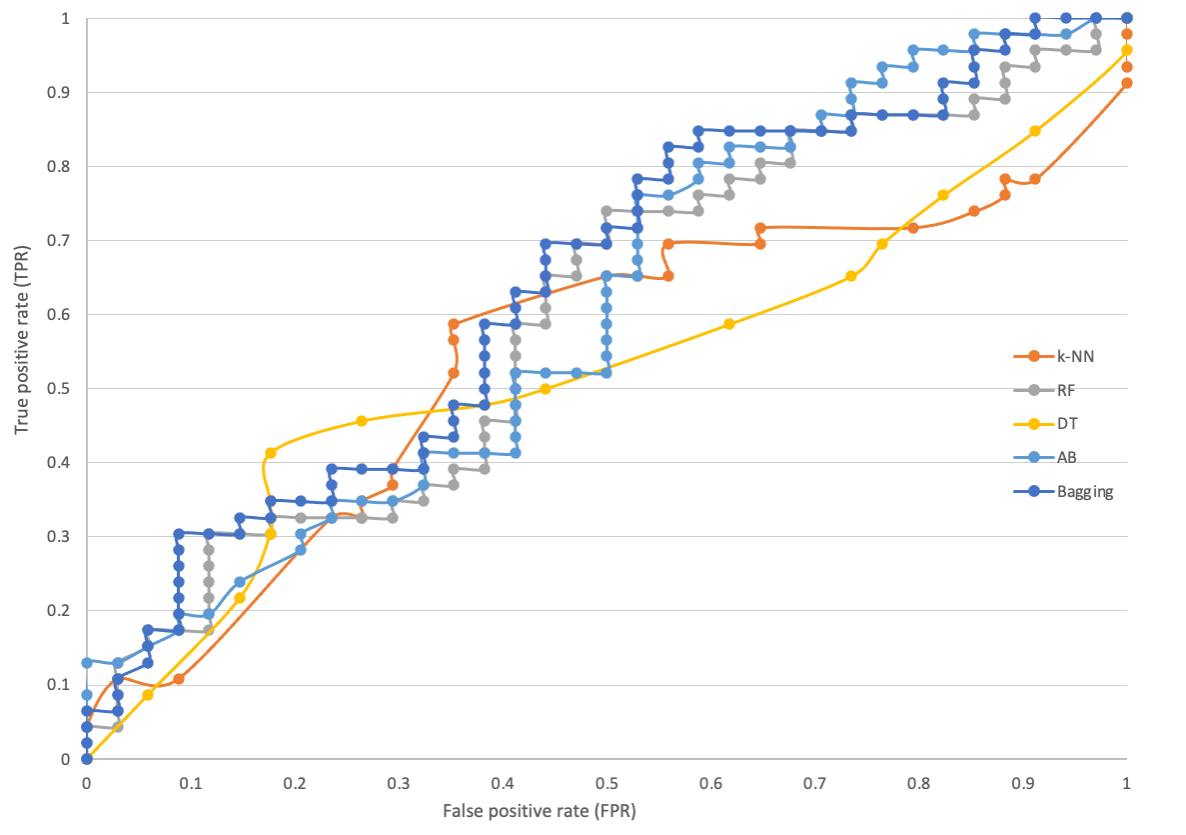
Receiver operating characteristic curves of all classifiers based on original data. AB: AdaBoost; DT: decision tree; k-NN: k-nearest neighbor; RF: random forest.

**Figure 4:**
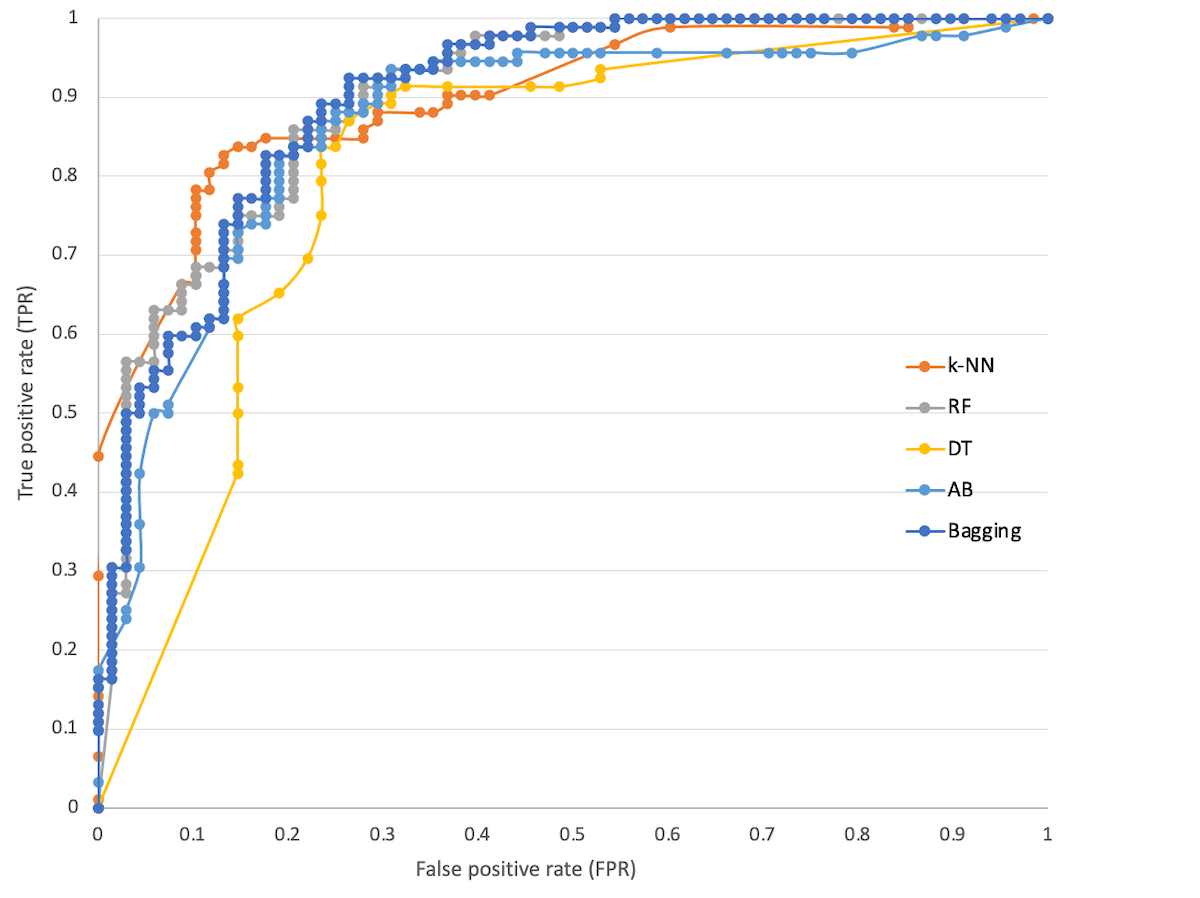
Receiver operating characteristic curves of all classifiers based on enriched data. AB: AdaBoost; DT: decision tree; k-NN: k-nearest neighbor; RF: random forest.

There is a clear difference between the values of all measures in Table 2 and Table 3 due to the feeding of additional records into the data set for data enrichment. The models’ performance based on the enriched data set has shown the values of precision, recall, and F-measure above 80% accuracy, except for a few values in Table 3. This is empirical evidence that populating the data set with additional records can increase the performance of the prediction models. Hence, Table 3 supports that these models can be used for maternity care decision making in identifying the mode of delivery before birth. Similarly, the models were analyzed using ROC curve evaluation [60]. ROC curves are highly useful for establishing the classifiers and envisioning their performance and are commonly used in health care decision making [61] because it visualizes the entire scenario of trade-off between recall and (1-specificity) across a set of cutoff points and is considered an effectual measure of inherent validity of a diagnostic test [62]. Moreover, as discussed in a previous study [9], ROC curves provide the percentage between precision and recall in which higher values of precision represent a low false-positive rate, which means that the classifier returns an accurate outcome, and the high values of recall showing a low false-negative rate, which means that the classifier returns positive outcomes. Figure 3 and Figure 4 show the ROC curves of all classifiers used for predicting the mode of delivery based on before and after data enrichment, respectively. The ROC curve has several advantages over single values of precision and recall in which one of its important benefits is that 2 or more diagnostic tests can be graphically compared at the same time in 1 graph [62]. Moreover, a curve that is nearer to the left upper corner shows the best accuracy of a classifier, while a curve closer to the lower right corner shows the worst [63]. In Figure 4, the curves closer to the left upper corner provide solid evidence, indicating that the accuracies of the classifiers used in the models based on enriched data are high. Therefore, these models are reliable and can be used for predicting the mode of delivery in the antenatal stage and can also be a part of the maternity care decision support system. Figure 3 shows ROC curves as middle lines, which are far away from the left upper corner compared to Figure 4; thus, Figure 4 is significantly more reliable.

## Discussion

### Principal Findings

**Figure 5:**
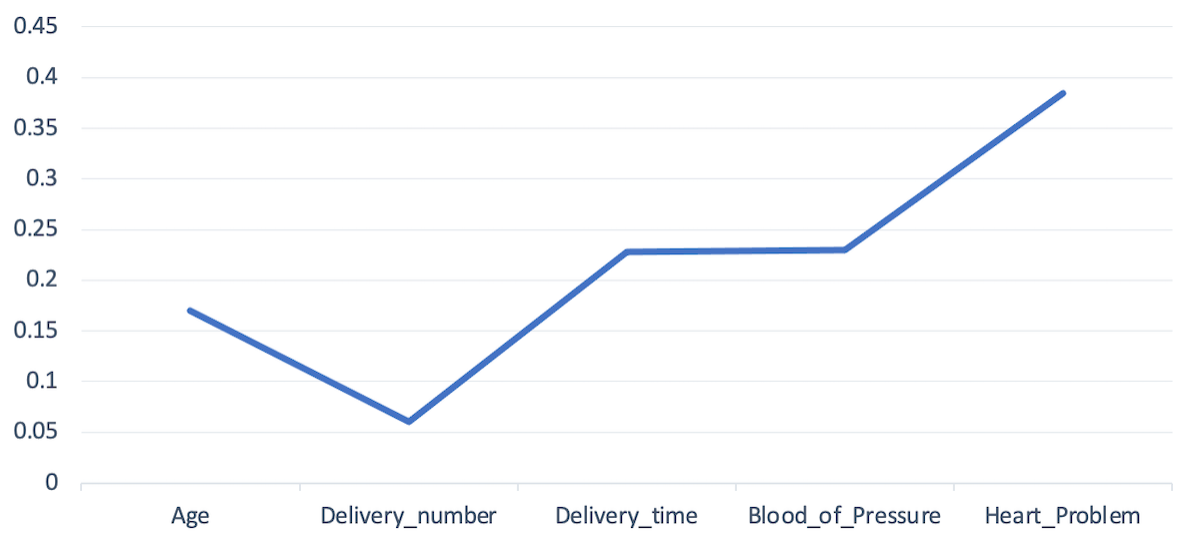
Correlation between attributes and class.

The outcomes in the above tables and figures show that the models’ performance in terms of accuracy, sensitivity, specificity, F-measure, and ROC curve is high when trained using the enriched data set compared to the measures achieved using the original data set. In particular, the outcomes shown in Table 1 and Table 3 and Figure 4 represent high model accuracies based on the enriched data set computed using various evaluation methods. All these models were evaluated using cross-validation, which is a commonly adopted method that is considered reliable for models’ evaluation in machine learning. In comparison, Amin and Ali [12] and Soleimanian et al [15] trained their models using the same data set (original) and achieved higher accuracy results than those in this study. The reason for achieving higher accuracy results was due to the optimistic method adopted for evaluation using the whole data set as a training set, which was not encouraged in several other studies such as that of Mitchell [48], Smith and Frank [45], and Brownlee [49]. Moreover, this study investigated the relationship of each attribute to its class. A correlation test was performed to identify factors influencing the mode of delivery. In this regard, the relationship of each attribute to its class was estimated. Figure 5 shows the correlation of each attribute to its class. The correlation of each attribute to its class is not high, but on closer investigation, this study concluded that the attribute “heart problem” is strongly correlated with class compared to other attributes, and this factor positively influences the mode of delivery. Thus, a patient with chronic heart-related issues may lead the obstetrician to a decision that is more favorable to c-section than normal delivery.

### Conclusion and Future Directions

This study investigated the mode of childbirth by pregnant women by using a machine learning approach. To this end, 5 classification models were trained in order to identify the optimal prediction model to assist obstetricians in decision making for the mode of delivery before birth. In the first part, the original data set was synthesized by populating its records based on the existing ones by using a standard machine learning approach referred to as SMOTE. In the second part, 5 machine learning models were trained based on the original and modified enriched data sets. The models that were trained using the enriched data set performed far better than those trained using the original data set in terms of accuracy, sensitivity, specificity, F-measure, and ROC. This clear difference in the results between the 2 sets of models was due to the increase of records in the original data set. In particular, for the model set trained with the enriched data set, k-NN outperformed the rest of the models with accuracy of 84.38%, while bagging, RF, DT, and AdaBoost showed accuracies of 83.75%, 83.13%, 81.25%, and 80.63%, respectively. Overall, the prediction models developed based on the enriched data set showed similar performances, and therefore the accuracy, sensitivity, specificity, F-measure, and ROC all indicate that these models should be used in the maternity care decision-making process as well as in assisting the obstetrician and midwife in making decisions about the mode of delivery before birth. The data set was artificially populated using a machine learning method. However, in future, if the same data set with the same features enriched with real clinical data will help identify more accurate results, the accuracy may be even more enhanced. The enriched data set in its current stage used in this study yields better results than the original data set, but this data set can become the best if the records are increased with real clinical data.

## Abbreviations

C-section: cesarean section
DT: decision tree
k-NN: k-nearest neighbor
RF: random forest
ROC: receiver operating characteristic
SMOTE: Synthetic Minority Oversampling Technique
